# Body and Mind at Cambridge

**Published:** 1891-03-28

**Authors:** Walter K. Sibley

**Affiliations:** 7, Harley Street, Cavendish Square, W.


### BODY AND MIND AT CAMBRIDGE.

Sir,—The writer of "Body and Mind at Cambridge," in the last issue of The Hospital, is decidedly antiquated with his information of the University. What he writes was true of Cambridge ten years ago and of Oxford to-day. He says, "Cambridge is still a school of mathematics first, and of natural science a long way after." This hardly accords with the figures as given in the Cambridge Calendar. Take, for instance, the numbers who take up the higher honours in mathematics and science—that is Part II. of the Tripos. For the last three years the figures are: Natural Science Tripos, Part II.—1888, 28; 1889, 28; 1890, 23. Mathematical Tripos, Part II.—1888, 11; 1889, 9; 1890, 11. That is, considerably more than double the number of science men take the second part of the Tripos to the Mathematical, and with the first part of the Tripos the numbers of science and mathematics are not so very dissimilar: Science Tripos Part I.—1888, 92; 1889, 104; 1890, 83. Mathematical Tripos, Part I.—1888, 121; 1889, 130; 1890, 124. I am almost certain I was told a year or so back by one of the examiners that there was actually more men in for Science Honours than for Mathematical, but that a larger percentage failed to get honours.—Yours faithfully,

Walter K. Sibley.

7, Harley Street, Cavendish Square, W.

